# Impact of automated thermal control on finishing pigs in semi-arid regions

**DOI:** 10.1007/s00484-025-03116-x

**Published:** 2026-01-08

**Authors:** Luana Barbosa Freire de Figueiredo, Pablo Teixeira Leal de Oliveira, Magno do Nascimento Amorim, Giovanni Antherreli Lima da Silva, Roniedson Fernandes da Silva Pequeno, Antônio Henrique Cardoso Sampaio Filho, Hércules Rodrigues Feitoza, Hugo Colombarolli Bonfá, Otoniel Cajuí Bonfim, Raquel Rafael de Freitas Silva, Deborah Cecília Trigueiro Custódio de Brito, Sílvia Helena Nogueira Turco

**Affiliations:** 1 Universidade Federal do Vale do São Francisco, Juazeiro, Bahia 48902-300 Brazil; 2 Instituto Federal do Sertão Pernambucano Campus Zona Rural, 56302-970 Petrolina, Pernambuco Brazil; 3 Escola de Agricultura Luiz de Queiroz da Universidade de São Paulo (Esalq/USP), São Paulo, 13418-900 Brazil

**Keywords:** Animal welfare, Microclimatic control, Precision livestock technologies, Swine production, Thermal comfort, Weight gain

## Abstract

Automation and environmental control offer solutions to enhance animal welfare and production efficiency in regions with adverse climates. This study aimed to develop a prototype for thermal environment control in finishing swine pens located in a semi-arid region and to evaluate its performance based on microclimatic traits, physiological responses, animal performance, and thermographic aspects of both the facility and the animals. The experiment involved thirty finishing gilts (Duroc × Large White crossbreeds), grouped by age and weight into three treatments (each with 10 animals per experimental unit). The automated thermal control treatments applied were: (1) no thermal control, (2) automatic activation of a micro-sprinkling system, and (3) automatic activation of micro-sprinkling combined with ventilation. Environmental conditions in all treatments failed to reach optimal thermal comfort levels for finishing pigs, due to the semi-arid characteristics of the region. However, the thermal control treatments reduced floor temperatures by approximately 3 °C compared to the treatment without thermal control. The micro-sprinkling treatment reduced the respiratory rate by 5.32 breaths/min and promoted an average daily weight gain of 0.26 kg/day compared to the control group. Additionally, the pigs’ body temperatures were significantly lower than those in the untreated group, indicating improved animal welfare. These findings demonstrate the efficacy of automated thermal control systems as a strategy to enhance swine production in semi-arid regions.

## Introduction

Brazil ranks as the fourth-largest pork producer and exporter globally (ABPA 2024). However, swine production is not equally distributed across all Brazilian regions. The Northeast, for instance, has lower production levels. In 2023, only 2.1% (43,901) of production systems in the Northeast followed a technology-intensive model, compared to 65.2% (1,377,313) in the South (ABCS 2024). The southern region of Brazil, the leading swine producer, processed 24.079 million swine in 2022, while the Northeast processed approximately 540,000, underscoring a production gap relative to the primary producing region (ABPA 2023).

The Brazilian Northeast is predominantly characterized by a semi-arid climate, which poses significant challenges for swine producers. The region’s low precipitation, low relative humidity, and high temperatures throughout most of the year negatively impact thermal conditions in animal facilities (Silva et al. 2023). These meteorological variables can lead to thermal stress, behavioral changes, and reduced productive and reproductive performance, ultimately lowering profitability (Oliveira et al. 2021).

Key indicators of thermal stress in production animals include physiological measures such as rectal temperature (Giannetto et al. 2025) and respiratory rate, behavioral responses, and productivity metrics (Oliveira et al.^2025^). Furthermore, Renaudeau and Dourmad (2022) emphasize that climate change represents an ongoing challenge for livestock production, potentially exacerbating future vulnerabilities in the swine sector. In this context, precision livestock farming (PLF) technologies enable effective monitoring of these parameters and facilitate targeted interventions to enhance production efficiency.

In response to environmental sustainability and animal welfare challenges, PLF technologies have emerged as a pathway toward sustainable livestock production (Papakonstantinou et al. 2024). These technologies enable simultaneous monitoring of physiological welfare indicators (Aragona et al. 2025), behavioral assessments (Amorim et al. 2024), and the integration of wearable sensors, environmental monitoring equipment, and remote sensing (Bernabucci et al. 2025). Such tools allow for individual and herd-level monitoring, supporting producers in making data-driven decisions (Lovarelli et al. 2024).

To maximize productivity, swine facilities must be designed to maintain thermal conditions within or near the ideal comfort range (Barnabé et al. 2020). PLF technologies optimize production by controlling environmental factors, particularly thermal conditions in housing systems (Norton et al. 2019; Zhang et al. 2021). However, as Trabachini et al. (2024) highlight, a critical challenge in PLF is the automation of microclimatic variable control to manage actuators effectively.

Knox (2025) emphasizes the need for modified housing for swine herds to meet animal welfare requirements. In this regard, the automation of thermal control represents one of these changes that promote animal welfare and may improve productivity indicators. Thus, the use of automated technologies and environmental control systems can enhance environmental conditions and provide thermal comfort for animals, especially in regions where conditions are adverse and where few studies have examined the influence of these technologies.

Therefore, this study aimed to develop a prototype for controlling the thermal environment in finishing pig pens in a semi-arid region and to evaluate its performance in terms of the microclimatic characteristics of the pens, physiological parameters, animal performance, and thermographic aspects of the facility and the animals.

## Materials and methods

The study was conducted on a commercial farm located in Casa Nova, Bahia, Brazil, from February to March 2023. The municipality was situated at 09º9’S latitude and 40º58’W longitude, at an altitude of 397 m. According to the Köppen classification, the climate is semi-arid, with an average annual temperature of 25.4 °C and an average annual precipitation of 485 mm. The activities were duly evaluated by the Ethics Committee on the Use of Animals and by the Ethics and Deontology Committee for Studies and Research of the Universidade Federal do Vale do São Francisco (UNIVASF), with approval number 0004/2,709,214, adhering to international standards on the ethical use of animals.

### Experimental characteristics

The commercial farm infrastructure included a barn with finishing pens for pigs, measuring 30.0 × 11.0 × 4.5 m (length, width, and height), with a galvanized steel roof at a 10% slope and supported a steel frame. The barn contained fourteen metal pens with partially slatted floors; three of these pens, each with a standard size of 4.0 × 3.8 m, were used for the experiment.

To avoid any potential sex-based interference, 30 Duroc x Large White crossbred females, with an average age of 120 days and an average weight of 70 kg, were used. The animals were in the finishing phase and were distributed by age and weight across three treatments with 10 replications, with each animal serving as an experimental unit. The animals’ diet was provided ad libitum and consisted of 75% corn bran, 23% soybean meal, and 2.0% growth core containing vitamins and minerals, provided for all treatments.

Three treatments were applied as follows: T1 – control pen, without thermal control; T2 – pen equipped with a micro-sprinkling system; and T3 – pen equipped with a combined micro-sprinkling and ventilation system. Daily sanitary and nutritional management of the animals followed the farm’s established protocol. Additionally, the development of the thermal controller used in the treatments to regulate the environment in the finishing pens will be discussed further.

### Thermal control system

The micro-sprinkling system consisted of two inverted ballet-type micro-sprinklers, one for micro-sprinkler treatment only and the other for micro-sprinkler and ventilation treatment. These 360° static micro-sprinklers (Nagura, São Carlos, SP, Brazil) were suspended 1.5 m above the facility floor, with an average flow rate of 77 L/h, a radius of 2.5 m, and an operating pressure of 0.5 m of water column (MWC).

The micro-sprinklers were installed with micro tubing and connectors attached to a 20 mm irrigation hose. The system operated intermittently, with 2 min of sprinkling activated when the micro-sprinkling pen temperature reached 27 °C. After this period, if the ambient temperature was at or below 27 °C, the system remained off. This system was connected to a 1000-liter reservoir of water.

The ventilation system consisted of an oscillating-wall axial fan (Ventisol^®^, New 60 cm, 147 W and maximum rotation speed of 1,300 rpm, Brazil), with three premium black blades, a 60 cm diameter, and a bivolt motor. The fan was mounted on a support column at a height of two meters above the floor, angled to cover the pen’s surface without lateral oscillation.

The fan had three speeds (low, medium, and high) and a power rating of 147 Watts, operating at the maximum speed of 3.88 m/s. Additionally, micro-sprinkler and ventilation treatment included the micro-sprinkler, which was activated simultaneously with the fan. The system alternated between 2 min of sprinkling and 18 min of ventilation when the air temperature in the ventilated pen reached 27 °C. The following topic will cover additional information regarding the development of this thermal controller in relation to obtaining ambient temperature values.

#### Development of the thermal controller

**Fig. 1:**
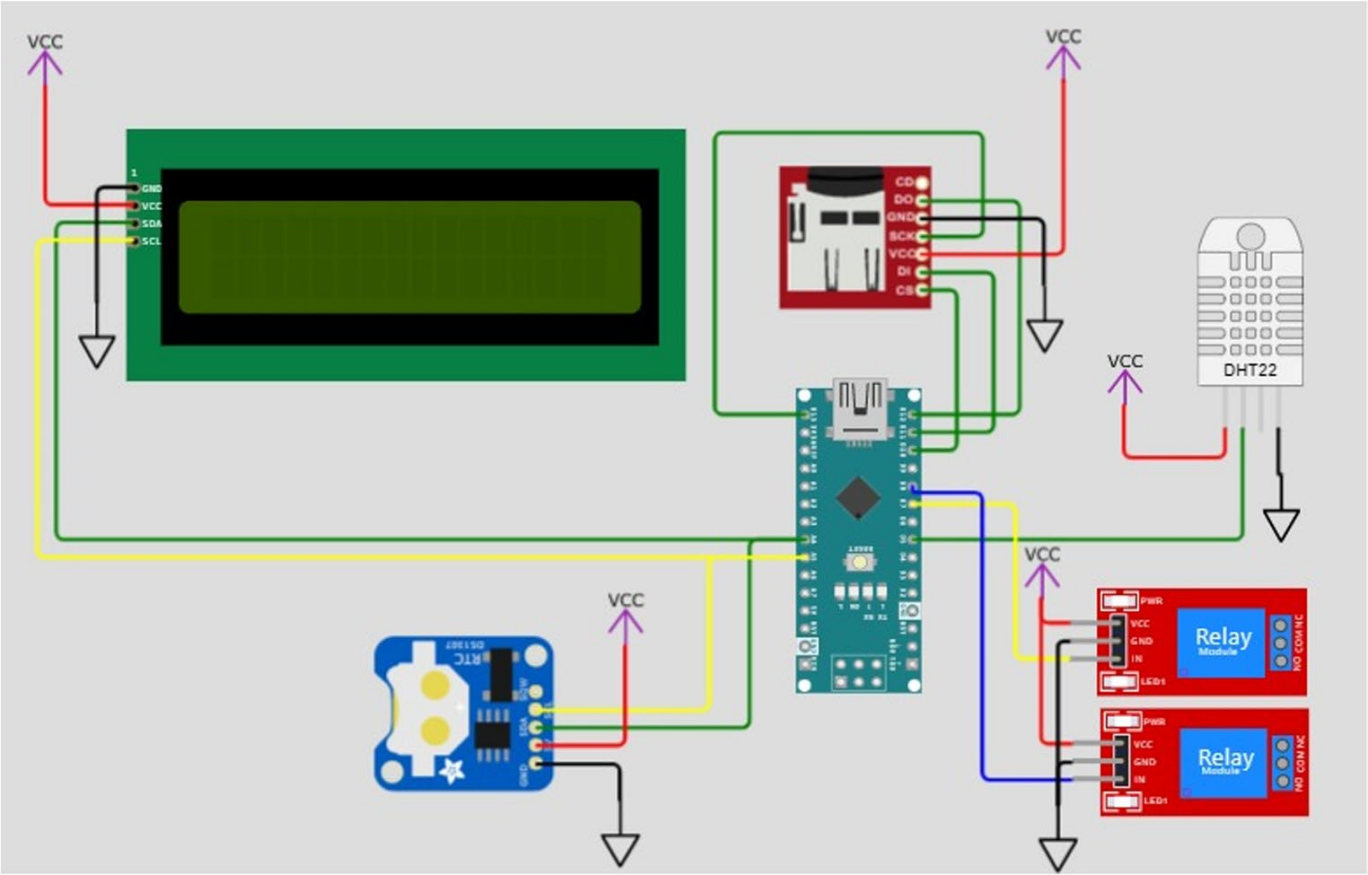
Schematic diagram of the controller

The prototype for thermal control was developed using an Arduino Nano microcontroller (ATmega328 Microcontroller, 20 Digital I/O Pins, 8 Analog Ports, 5 V Operating Voltage, Maximum Current of 40 mA, and 32 KB Flash Memory, Monza, Italy) and a temperature and humidity sensor DHT11 (humidity measurement range: 20 to 90% RH, humidity accuracy: ±5% RH, temperature measurement range: 0 °C to 50 °C, error margin: ±4% RH | ±2 °C.). The system was connected to a real-time clock module (DS3231) and a non-volatile memory device containing an 8 GB SD card for experimental data storage (air temperature and relative humidity), which was recorded every minute throughout the experiment (30 days). The sensor was installed at a height of 1.5 m, and a calibration curve was performed to convert the measured values to actual temperature. A 16 × 2 LCD display was connected to an L2C Serial Module, which was also linked to the DHT11 sensor and a two-channel relay (Fig. 1).

**Fig. 2:**
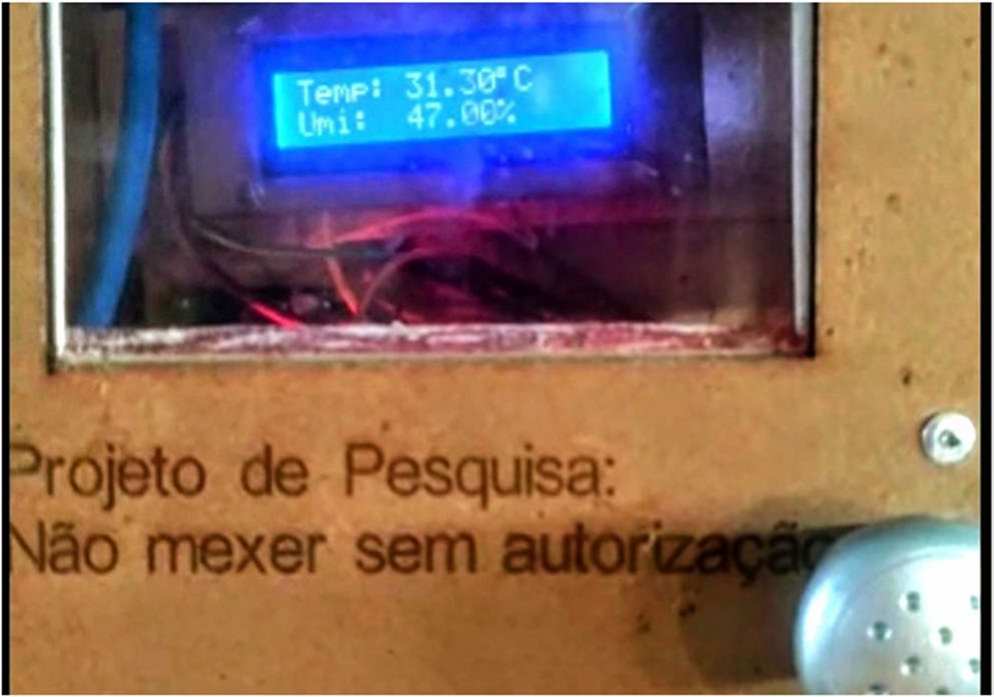
LCD display showing experiment values

The prototype for thermal control was programmed to meet the desired functionality of a temperature control system for air-conditioned pens. Programming was performed using the Arduino IDE software. The system started operating when the power button was pressed and indicated on the 16 × 2 LCD screen (Fig. 2) whether it was working. In addition, this configuration allowed the collection and recording of air temperature and relative humidity data using the DHT11 sensor.

The controller was set to activate the micro-sprinkling and micro-sprinkling + ventilation systems when the air temperature sensor recorded readings > 27 °C, and it deactivated at readings ≤ 27 °C. Due to the high ambient temperatures characteristic of the Brazilian semi-arid region where the experiment took place, the upper critical temperature of 27 °C was chosen, in line with Diniz (2023). The micro-sprinkling system was equipped with a ½ horsepower motor pump, while the ventilation system used an industrial fan.

#### Thermal controller validation

The validation of the developed thermal controller was based on a comparative analysis between the data recorded by the system (DHT11 sensor) and those obtained from commercial data loggers (Onset HOBO^®^ TEMP/RH/2 ext channels; accuracy of ± 3% for RH and ± 1 °C for temperature, United States), all with calibration certificates.

This analysis was performed using statistical error indices, namely the bias (Eq. 1), the root mean square error (RMSE) (Eq. 2), and the coefficient of determination (R²) (Eq. 3), calculated according to the following equations.

1 $$\:Bias=\:\frac{1}{n}\:\sum\:_{i=1}^{n}(Pi-Oi)$$

2 $$\:RMSE=\:\sqrt{\frac{1}{n}\sum\:_{i=1}^{n}{(Pi-Oi)}^{2}}$$

3 $$\:{R}^{2}=1-\:\frac{\sum\:_{i=1}^{n}{(Oi-Pi)}^{2}}{\sum\:_{i=1}^{n}{(Oi-\:\overline{O})}^{2}}$$

Where 𝑃𝑖 represents the predicted values (measured by the thermal controller), 𝑂𝑖 the observed values (recorded by the data logger), $\:\overline{O}$ the mean of observed values, and 𝑛 the total number of observations.

### Environmental parameters analyzed

To assess the thermal environment, the following data were collected daily over the 30-day experiment: dry bulb temperature (Tbs), dew point temperature (Tpo), relative humidity (RH), and black globe temperature (Tgn). Measurements were taken every 15 min using data logger sensors (Onset HOBO^®^ TEMP/RH/2 ext channels; accuracy of ± 3% for RH and ± 1 °C for temperature, United States), totaling 96 recordings per day. These sensors were suspended 1.5 m above the facility floor, with two sensors per experimental pen, resulting in six data loggers within the barn and one outside.

The following thermal comfort indices were calculated based on the microclimatic data obtained: Temperature and Humidity Index (THI), Black Globe Temperature and Humidity Index (BGHI), and Enthalpy (H). These indices are defined by Eqs. 4, 5, and 6.

The Temperature and Humidity Index (THI) was calculated using the equation proposed by Thom (1959):

4 $$\:\mathrm{T}\mathrm{H}\mathrm{I}=\mathrm{T}\mathrm{a}\mathrm{r}+\mathrm{0,36}\mathrm{T}\mathrm{p}\mathrm{o}+\mathrm{41,5}$$

Where Tar is air temperature (°C), and Tpo is dew point temperature (°C).

The Black Globe Temperature and Humidity Index (BGHI) was determined using the equation by Buffington et al. (1981):

5 $$\:\mathrm{B}\mathrm{G}\mathrm{H}\mathrm{I}=\mathrm{T}\mathrm{gn}+\mathrm{0,36}\mathrm{T}\mathrm{p}\mathrm{o}+\mathrm{41,5}$$

Where Tgn is black globe temperature (°C), and Tpo is dew point temperature (°C).

Enthalpy (h) was calculated using the equation proposed by Rodrigues et al. (2011):

6 $$\:h=\mathrm{1,006}.Tar+\frac{RH}{PB}{10}^{\left(\frac{\mathrm{7,5}Tar}{\mathrm{273,3}+Tar}\:\right)}.(\mathrm{71,28}+\mathrm{0,052}Tar)$$

Where Tar is air temperature (°C), RH is relative humidity (%), and PB is local barometric pressure (mmHg).

### Physiological parameters of animals analyzed

Physiological parameters were measured on the 7th, 14th, 21 st, and 28th days of the experiment. Measurements included respiratory rate (RR), surface skin temperature (ST), and rectal temperature (RT) at 8 am and 3 pm. Measurements were conducted at these times to capture both thermally mild conditions and critical heat periods representative of the semiarid region’s climatic variation. The 8 am timepoint was selected as the earliest feasible data collection window considering commercial farm operations, while 3 pm was chosen to represent peak ambient temperatures (near the daily maximum at ~ 2 pm) while maintaining adequate intervals between measurements. The trained operator followed established protocols for data collection. Notably, animals were acclimated to both the operator and measurement procedures, eliminating the need for physical restraint. Consequently, no containing techniques were employed during data collection, as the animals remained calm throughout the process.

Surface skin temperature was determined using an infrared thermometer (Digital Infrared Thermometer ST/600, −60 °C to + 500 °C, INCOTERM, China) at a distance of 20 cm from each of the measured points (Amaral et al. 2014) on six specific body locations (forehead, shoulder, loin, flank, ham, and hock), with the average calculated subsequently.

Rectal temperature was measured with a veterinary clinical thermometer graduated up to 44 °C, inserted 5.0 cm into the rectum of each animal for two minutes (RT; °C). Respiratory rate (RR) was determined by counting flank movements over 15 s and multiplying the result by four to obtain breaths per minute (RR; breaths min⁻¹).

### Performance analysis

The animals’ initial body weight (IBW) was recorded before feed and water were withheld. Subsequent weigh-ins were conducted every seven days until the end of the 30-day experimental period, yielding the final body weight (FBW). Average daily gain (ADG) was calculated using the Eq. 7.

7 $$\:ADG\:\left(kg\right)=\left(FBW-IBW\right)/\:30\:$$

Animals were weighed using a metal digital scale with a capacity of 500 ± 0.100 kg. To minimize disruption to farm operations, they were not fasted prior to weigh-ins from the start to the end of the experiment. Due to the use of only three pens and adherence to regular farm protocols, individual feed intake measurements were not possible, which restricted the calculation of feed conversion ratios.

### Thermographic characteristics of the animals and the facility

**Fig. 3:**
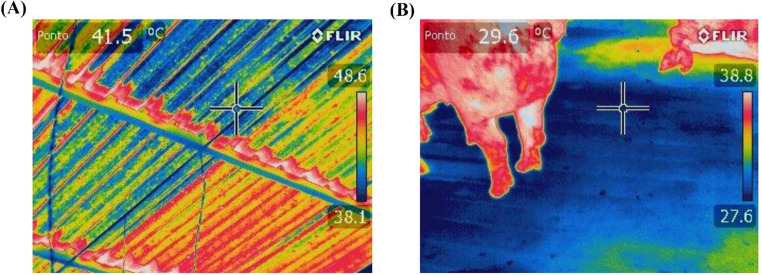
Example of a thermographic image of the roof (**A**) and the floor (**B**) of the facility

Thermographic images were acquired for five animals as well as for the facility’s roof and floor (Fig. 3) on the 7th, 14th, 21 st, and 28th days of the experiment for each treatment. All images were captured at 4 pm, with the same animals being photographed on each day.

The thermographic images were obtained using a FLIR-T62101 camera (display resolution of 320 × 240 and thermal sensitivity of 40 mK, United States). Images were captured at a distance of 0.25 m from the target, with the emissivity set at 0.98, following the manufacturer’s recommendations for temperature measurement in biological tissues (Possagnolo et al. 2024). The camera’s focus was automatic, and a series of images were taken to select the most suitable one. Additionally, during image processing, the regions of interest were later selected and evaluated using the pointer tool. The images were then transmitted and processed with Flir Tools software, which provided thermographic profiles for both the animals and the facility, yielding maximum, average, and minimum temperature values.

**Fig. 4:**
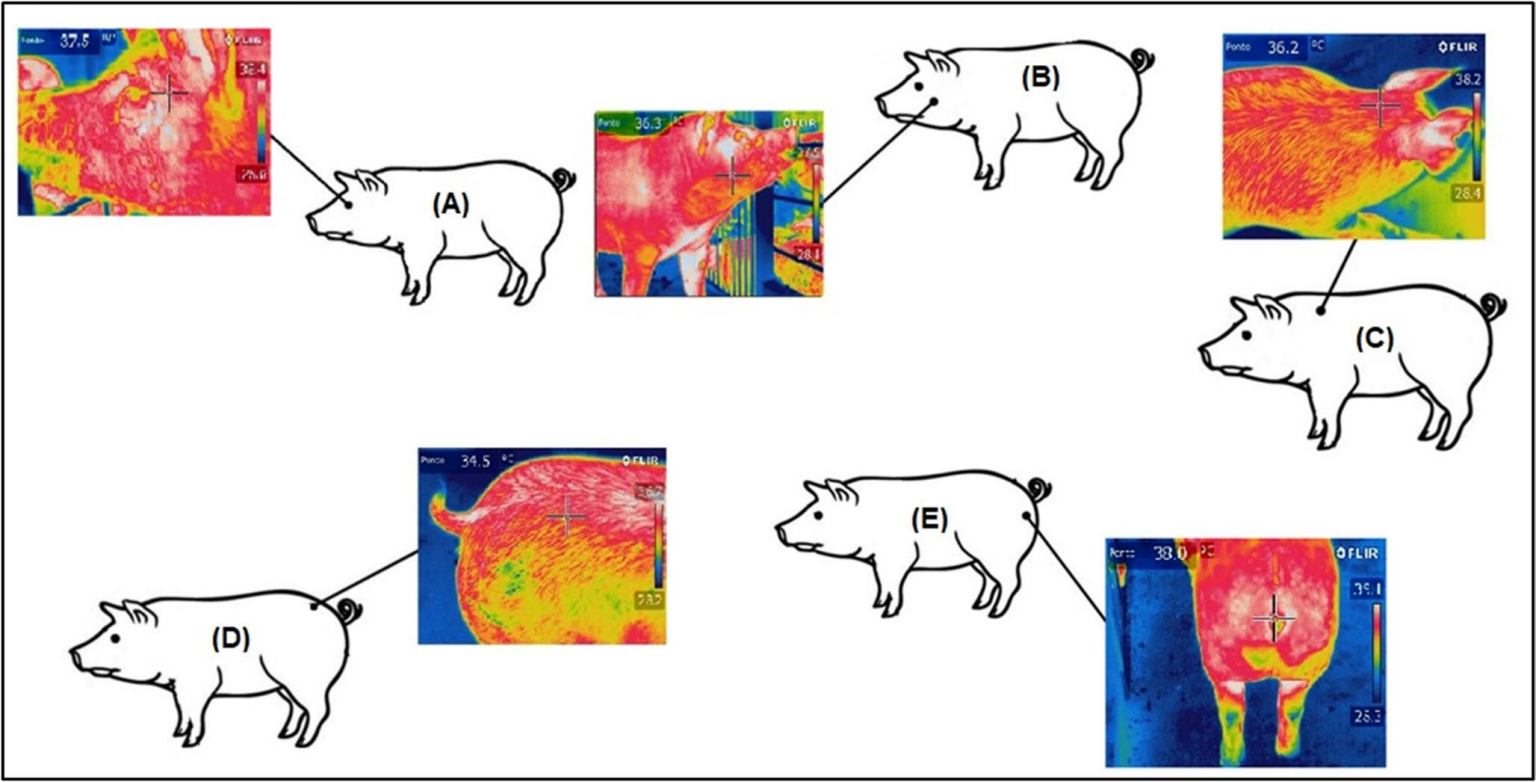
Eye region (**A**), cheek region (**B**), cervical-dorsal region (**C**), caudal-dorsal region (**D**), and posterior region (**E**)

The specific data collection points on the pigs included the eye region, cheek region, cervical-dorsal region, caudal-dorsal region, and posterior region (Fig. 4).

### Statistical analysis

Environmental parameters were analyzed using a randomized block design (RBD) with three treatments (no thermal control, micro-sprinkling, micro-sprinkling + ventilation) and blocks representing the 30-day evaluation period. Physiological and performance parameters were analyzed in a factorial scheme (3 × 2) within an RBD, with the treatments as the first factor and the sampling times (8 am and 3 pm) as the second factor. Blocks represented the different days of data collection (7th, 14th, 21 st, and 28th days), and experimental units were the 10 animals per treatment. Thermographic parameters were analyzed using the RBD with the different collection days representing the blocks. All statistical analyses were performed using R software. The Shapiro-Wilk test was used to assess residual normality, while Levene’s test was used to evaluate homogeneity of variances. When significant differences were found, analysis of variance was followed by Tukey’s test (*p* < 0.05), along with regression analyses for environmental data.

## Results

**Fig. 5:**
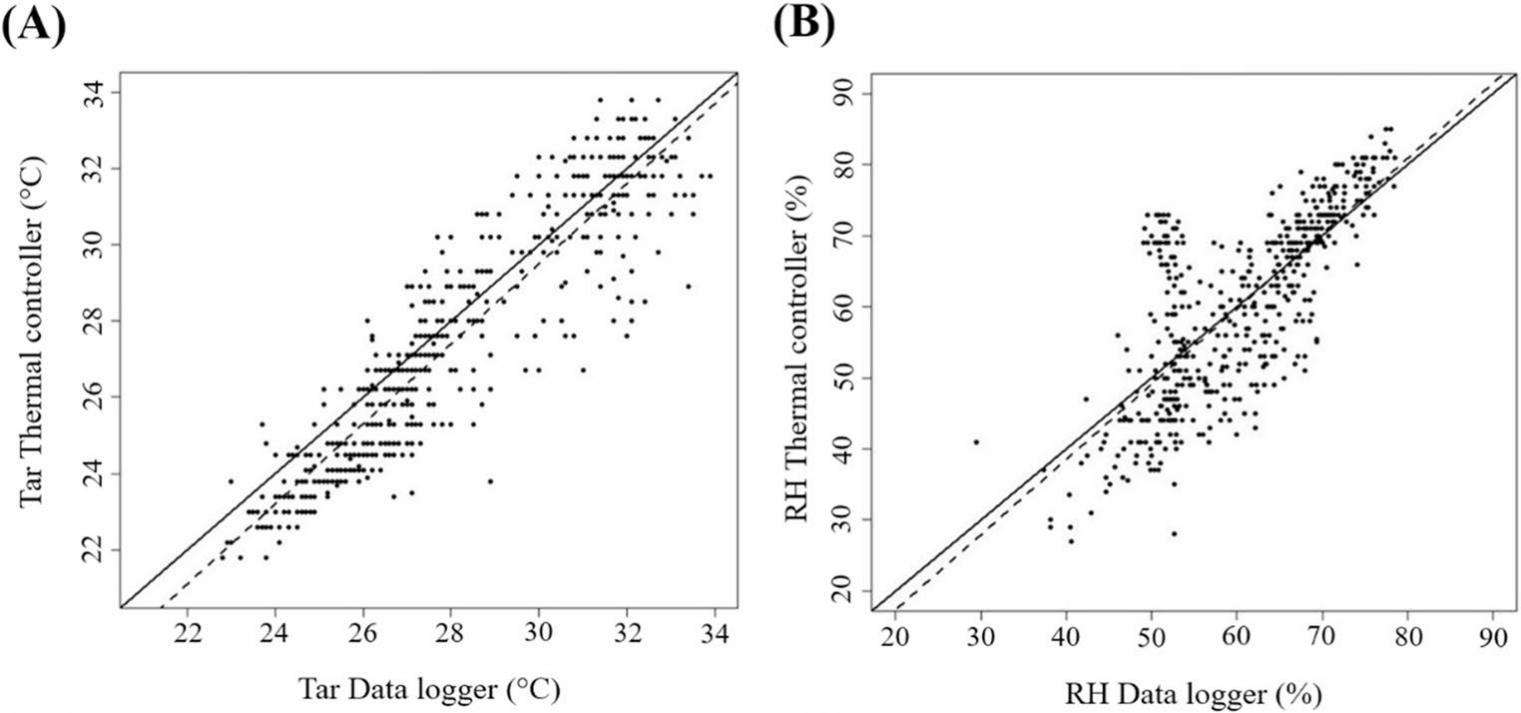
Scatter plots of air temperature (**A**) and relative humidity (**B**) between the thermal controller and the data logger

Figure 5 shows the comparison between air temperature and relative humidity recorded by the thermal controller and the data logger.

Comparative metrics between the thermal controller and the data logger are presented in Table 1. For air temperature (TA), the bias was negative (−0.59), indicating a slight underestimation relative to the data logger. The RMSE value of 1.38 reflects good precision of the measurements obtained by the controller. The coefficient of determination (R² = 0.83) indicates a strong correlation between the recorded values, with low dispersion among data points. For relative humidity (RH), the bias was also negative (−0.28) and close to zero, suggesting good agreement between sensors. However, the RMSE value was higher (8.30), resulting in a lower coefficient of determination (R² = 0.58), indicating greater variability in humidity data compared with temperature measurements.

**Table 1:** Statistical indicators comparing the sensor connected to the thermal controller and the HOBO^®^ data logger for air temperature (Tar) and relative humidity (RH)

Indices	Air temperature	Relative Humidity
Bias	−0.59	−0.28
RMSE	1.38	8.30
R²	0.83	0.58

Key environmental parameters were evaluated across the three treatments, examining thermal comfort indices derived from the data loggers (Table 2).

**Table 2:** Mean values for air temperature (Tar), relative humidity (RH), enthalpy (h), black globe temperature and humidity index (BGHI), and temperature and humidity index (THI) obtained from data loggers throughout the day across the experimental period

Variables	Treatments			SEM	*p*-value
	Without thermal control	Micro-sprinkling	Micro-sprinkling with Ventilation		
Tar (°C) ^*^	28.41 a	27.41 b	28.19 a	0.095	< 0.0001
RH (%)	65.77	65.35	66.09	0.358	0.3507
h ^*^	71.90 a	68.35 b	71.49 a	0.171	< 0.0001
BGHI ^*^	76.64 a	75.79 b	76.79 a	0.110	< 0.0001
THI ^*^	77.18 a	75.79 b	76.94 a	0.100	< 0.0001

^*^Different letters indicate statistical significance (*p* < 0.05) across columns;* SEM *standard error of the mean

For the thermal environment data of the pig finishing pens, significant differences were observed in mean air temperature (*p* < 0.05), with micro-sprinkling exhibiting lower air temperatures compared to other treatments. No significant differences were observed in mean relative humidity across treatments. However, for *h*, BGHI, and THI, significant differences were noted between treatments, with the micro-sprinkling group demonstrating the lowest mean values for environmental parameters.

**Fig. 6:**
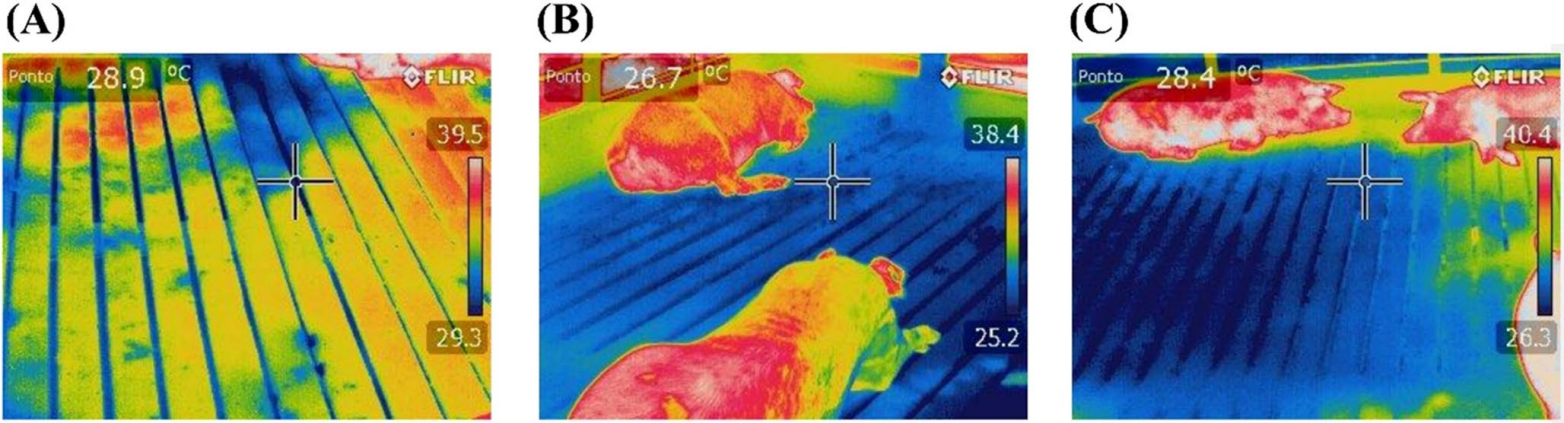
Example of the thermographic image of the floor for the treatments Without thermal control (**A**), Micro-sprinkling (**B**), and Micro-sprinkling with Ventilation (**C**)

Table 3 presents the maximum, mean, and minimum temperature values for the roof and floor surfaces of the pens across the three treatments. It can be observed that, in no case, were there significant differences in the roof temperatures among the treatments (*p* > 0.05). Conversely, the automated thermal control significantly influenced floor temperatures, with treatments micro-sprinkling and micro-sprinkling with ventilation exhibiting lower temperatures compared to the treatment without thermal control (*p* < 0.05). Figure 6 provides an example of thermal images of the floors under the different treatments, visually indicating that the control treatment was warmer.

**Table 3:** Values of maximum, mean, and minimum temperature for the roof and floor surfaces of the pens

	Treatments			SEM	*p*-value
	Without thermal control	Micro-sprinkling	Micro-sprinkling with Ventilation		
Maximum temperature (°C)					
Floor ^*^	31.25 b	27.82 a	27.85 a	1.137	0.041
Roof	42.60	41.43	40.78	0.534	0.499
Mean temperature (°C)					
Floor ^*^	30.30 b	27.00 a	26.70 a	1.153	0.023
Roof	38.50	38.43	38.45	0.354	0.664
Minimum temperature (°C)					
Floor ^*^	28.93 b	25.63 a	25.43 a	1.134	0.017
Roof	35.80	35.63	37.08	0.457	0.591

^*^Different letters indicate statistical significance (*p* < 0.05) across columns; *SEM *standard error of the mean

Regarding physiological variables, no interaction was found between sampling times and treatments, so they were analyzed separately. For mean rectal temperature, no significant differences were observed between the sampling times (*p* > 0.05). However, for surface temperature and respiratory rate, significant differences were found for these two variables (*p* < 0.05) between the two sampling times (Table 4).

**Table 4:** Mean values for surface temperature, rectal temperature, and respiratory rate at 8 am and 3 Pm

Timetables	Surface Temperature (°C) ^*^	Rectal Temperature (°C)	Respiratory Rate (mov/min)
8 am	34.43 a	39.35	12.46 a
3 pm	35.80 b	39.39	16.65 b
SEM	0.400	0.059	1.057
p-value	0.0191	0.6723	0.0075

^*^ Different letters indicate statistical significance (*p* < 0.05) across rows; *SEM* standard error of the mean

Table 5 provides the physiological and performance data for pigs across the three treatments. No significant differences were observed in mean surface and rectal temperatures between treatments (*p* > 0.05). However, for respiratory rate, significant differences were found, with animals in the micro-sprinkling and micro-sprinkling with ventilation groups showing lower respiratory rates compared to the group without thermal control (*p* < 0.05). As for performance data, average daily weight gain was higher for pigs in the micro-sprinkling-only treatment compared to the other treatments.

**Table 5:** Mean values for surface temperature, rectal temperature, respiratory rate, and average daily weight gain across the treatments

Treatments	Surface Temperature (°C)	Rectal Temperature (°C)	Respiratory Rate (mov/min) ^*^	Average Daily Weight Gain (kg/day) ^*^
Without thermal control	35.39	39.50	17.42 b	0.69b
Micro-sprinkling	35.39	39.44	12.10 a	0.95a
Micro-sprinkling with Ventilation	34.56	39.16	14.15 ab	0.77b
SEM	0.490	0.073	1.295	0.045
p-value	0.3966	0.0539	0.0195	0.0003

^*^Different letters indicate statistical significance (*p* < 0.05) across rows; *SEM *standard error of the mean

Table 6 presents the maximum, mean, and minimum temperature values across treatments for the eye, cheek, cervical-dorsal, caudal-dorsal, and posterior regions of the animals. Significant differences (*p* < 0.05) in maximum temperature were observed only in the cervical-dorsal region, with the highest value recorded in the treatment without thermal control. For average temperature, significant differences were found in the cervical-dorsal and caudal-dorsal regions, where the treatment without thermal control also exhibited the highest values. Similarly, this treatment showed significantly higher mean temperatures in both the caudal-dorsal and posterior regions.

**Table 6:** Values of maximum, mean, and minimum temperature for the eye, cheek, cervical-dorsal, caudal-dorsal, and posterior regions under different treatments: without thermal control, Micro-sprinkling, and Micro-sprinkling with ventilation

	Treatments			SEM	*p*-value
	Without thermal control	Micro-sprinkling	Micro-sprinkling with Ventilation		
Maximum temperature (°C)					
Eye	37.93	37.24	37.04	0.269	0.356
Cheek	38.31	37.90	38.22	0.124	0.577
Cervical-dorsal ^*^	38.92 b	38.00 a	38.45 ab	0.264	0.003
Caudal-dorsal	39.53	38.82	38.69	0.259	0.057
Posterior	39.77	39.37	39.43	0.124	0.153
Mean temperature (°C)					
Eye	37.03	36.19	35.77	0.368	0.305
Cheek	37.10	36.13	36.63	0.280	0.218
Cervical-dorsal ^*^	37.55 b	36.51 a	37.05 ab	0.300	0.037
Caudal-dorsal ^*^	38.36 b	37.06 a	37.41 ab	0.388	0.012
Posterior	38.13	37.14	37.28	0.311	0.076
Minimum temperature (°C)					
Eye	35.74	34.91	34.40	0.389	0.343
Cheek	35.03	33.79	33.67	0.434	0.308
Cervical-dorsal	35.67	33.52	34.27	0.628	0.052
Caudal-dorsal ^*^	35.81 b	33.18 a	34.27 ab	0.763	0.011
Posterior ^*^	33.72 b	31.57 a	31.27 a	0.772	0.018

^*^Different letters indicate statistical significance (*p* < 0.05) across columns; *SEM *standard error of the mean

## Discussion

The Tar values recorded by the thermal controller tended to slightly underestimate those from the data logger, showing greater dispersion above 27 °C and a regression line positioned below the 1:1 reference, resulting in a negative bias. For RH, a wider dispersion and similar underestimation were observed, with most points falling below the ideal line, which explains the lower R² and higher RMSE compared with Tar. These discrepancies are mainly attributed to the controller’s programming, which prioritizes temperature as the primary control variable, while RH serves as a secondary indicator. Additionally, the DHT11 sensor used in the prototype has lower accuracy and resolution, particularly for humidity measurements, contributing to the observed deviations (Saputro and Yantidewi 2021).

The recommended air temperature range for finishing pigs is between 12 °C and 18 °C, with an upper critical limit of 27 °C (Oliveira et al. 2016). In this study, the micro-sprinkling treatment was closest to this upper limit, averaging 27.41 °C, while other treatments exceeded 28 °C. RH for pigs in the growing and finishing phases averages 70%, with critical thresholds below 60% and above 80% (Ferreira, 2015). None of the treatments exceeded these RH comfort limits (Table 1).

Regarding thermal comfort indices, the *h* value for the micro-sprinkling treatment averaged 68.35 kJ/kg of dry air, within the comfort range of 60.4–68.6 kJ/kg of dry air for finishing pigs (Castro Júnior and Silva 2021). In contrast, the treatments without thermal control and micro-sprinkling combined with ventilation exceeded 71 kJ/kg of dry air, indicating thermal discomfort due to limited evaporative heat dissipation (Gomes et al. 2021).

A significant difference (*p* < 0.05) was observed in the BGHI across treatments. The micro-sprinkling treatment yielded the lowest average (75.79), while the treatments without thermal control and micro-sprinkling with ventilation showed similar values (76.64 and 76.79, respectively). All treatments exceeded the comfort threshold of 74 for finishing pigs (Alves et al. 2023). Oliveira et al. (2016) reported BGHI values below 72 in Pirassununga/SP, while Barnabé et al. (2020) found values exceeding 76 in non-climatized pens in Pernambuco’s semi-arid region, with climatized pens recording lower values (72.2), consistent with this study’s findings.

The micro-sprinkling treatment also recorded the lowest average THI. Mellado et al. (2018) classified THI values between 74 and 78 as indicative of mild heat stress, with values below 74 considered comfortable and above 82 indicating severe stress. The THI values in this study suggest mild heat stress, remaining below the critical upper limit of 85 (Moi et al. 2014), though the comfort threshold (THI < 74) was not achieved.

The micro-sprinkling treatment consistently showed the lowest values for most microclimate variables. Previous studies suggest that pigs prefer micro-sprinkling systems for heat exchange, spending more time in such environments (Godyń et al. 2020; Jeppsson et al. 2021). This preference may be attributed to the system’s location and orientation, influenced by breezes from a nearby river driven by prevailing southeastern winds (Araújo Júnior et al. 2019).

Ventilation, natural or artificial, combined with sprinklers or misting systems can enhance thermal comfort for finishing pigs (Santos et al. 2012). Although thermal control did not affect roof temperatures, floor temperatures were lower in controlled environments. The region’s low relative humidity (Cunha et al., 2015) may have further favored the micro-sprinkling treatment.

Higher values at 3 pm, compared to 8 am, reflect typical diurnal temperature variations. In semi-arid regions, high solar radiation due to proximity to the Equator makes noon the most critical period. However, Santos et al. (2011) identified 2–3 pm as the hottest times, due to heat absorption and transmission in facilities.

Pigs, as homeothermic animals, maintain a body ST between 33 °C and 35 °C (Gomes et al. 2021), consistent with this study’s findings. Alves et al. (2023) reported higher ST values (37.51–38.45 °C) in growing pigs under different climate control systems. ST varies by breed, reproductive status, environmental factors, and metabolic rate (Nazareno et al. 2012; Sørensen and Pedersen, 2015). Differences between ST and ambient temperature facilitate heat dissipation and are influenced by climate control systems (Rigo et al. 2019). In this context, environmental conditions significantly affect livestock body temperature (Giannetto et al. 2022), which may impact factors such as feed intake and weight gain (Giannetto et al. 2025).

No significant difference (*p* > 0.05) in RT between treatments suggests that animals did not experience extreme heat stress, remaining within their thermoneutral zone, close to the 39.3 °C comfort threshold (Kiefer et al. 2010). Lykhacha et al. (2022) reported similar RT values (39.2 °C) for finishing pigs, within the acceptable range (< 39.5 °C).

The RR for growing pigs typically ranges between 29.1 and 32.7 breaths per minute, higher than the values observed in this study. Lykhacha et al. (2022) observed RR averaging 20 breaths per minute in cooler conditions (≤ 8 °C). For adult pigs, normal RR ranges from 20 to 40 breaths per minute (Pereira et al. 2019), with values in this study falling within this range. Gomes et al. (2021) reported higher RR values in Pernambuco’s semi-arid region under various climate control systems.

RT and RR are influenced by factors such as age, breed, time of day, feed or water intake, ambient temperature, wind speed, season, and facility location (Perissinotto et al. 2009). Although high air temperatures can elevate RT and RR (Silva et al. 2023), this was not observed in this study, as values remained within comfort ranges (Souza et al. 2020). Silva et al. (2023) and Rigo et al. (2019) emphasize that heat stress beyond comfort zones negatively impacts physiological responses, which was not evident here.

Infrared thermography has been used to detect sick animals, identify clinical signs, and monitor fever or elevated body temperatures (Sørensen et al., 2015; McManus et al. 2016). Aragona et al. (2025) emphasized that changes in body temperature represent a physiological defense response to disruptions in homeostasis. Lower temperatures in specific body regions suggest that thermal control measures, such as spraying and ventilation, can reduce body temperature (Alves et al. 2024). Ricci et al. (2019) highlighted its utility in identifying surface temperature variations, aiding facility evaluation and animal welfare assessment. Alves et al. (2023) reported similar findings in growing pigs under control, evaporative cooling, and forced ventilation treatments, with the group without thermal control showing the highest dorsal-caudal temperatures, consistent with the findings in this study (Table 5).

The ideal ADWG for finishing pigs is 0.89–1.1 kg/day (Araújo 2021). The group treated with micro-sprinkling achieved a higher ADWG (0.95 kg/day), exceeding the expected 0.850 kg/day for the growing and finishing phases (Dias et al. 2011), highlighting the treatment’s positive impact on performance. In contrast, both the non-thermally controlled treatment and the combined micro-sprinkling + ventilation treatment showed lower average daily weight gains compared to the target range. One potential explanation for the underperformance of the micro-sprinkling + ventilation treatment is the fan speed, which operated at maximum capacity and may have dispersed water droplets before sufficient heat absorption could occur, thereby reducing cooling efficiency. While micro-sprinkling alone likely maintained an optimal balance between humidity and evaporative cooling, its combination with forced ventilation appears to have disrupted this equilibrium, resulting in excessive evaporation without meaningful thermal benefits.

It should be noted that the proposed system remained operational throughout nearly the entire experimental period, with only a single day of downtime for microcontroller programming adjustments. This underscores the practical viability of such technologies for implementation in production environments to enhance animal welfare.

To ensure the reliability of the developed thermal control system, its sensors were validated against a calibrated commercial data logger (Onset HOBO^®^ TEMP/RH/2 ext channels). Statistical indices (Bias, RMSE, and R²) were calculated to compare temperature and relative humidity readings between both devices. The results showed strong agreement (R² = 0.83 for air temperature and 0.58 for relative humidity), with low bias values (–0.59 and − 0.28, respectively), indicating that the controller provided accurate and consistent measurements under field conditions. This validation confirms the technical feasibility of the proposed prototype for real-time environmental monitoring and automated climate control in swine facilities. Although economic feasibility was not assessed in the present study, future research will include cost analysis and long-term performance evaluation to support large-scale implementation.

This trial was conducted in a commercial swine facility under standard management and nutritional practices, which precluded individual feed intake measurements and feed conversion ratio calculations. Furthermore, only three pens were available for experimental use, preventing statistical analysis of pen-level feed efficiency. Future studies should incorporate more pens to properly evaluate feed efficiency across different thermal management strategies and enable precise feed conversion ratio determination.

Despite initial resistance among producers to adopting technologies that promote animal welfare (Moreira et al. 2024), this study demonstrates the potential of simple and scalable thermal control systems in semi-arid regions, as evidenced by improvements in swine weight gain. Among the practical applications of this research, it is noteworthy that the micro-sprinkling system can be adapted for use in other semi-arid regions, promoting greater thermal comfort and productivity. Additionally, the combination of natural or artificial ventilation with micro-sprinkling can optimize energy use while maintaining thermal comfort for the animals. Finally, educating producers about the benefits of thermal control technologies may encourage their adoption, improving both swine welfare and productivity.

## Conclusion

The micro-sprinkling treatment yielded the best results for physiological parameters, environmental indices, and daily weight gain. However, none of the treatments provided ideal thermal comfort conditions for finishing pigs due to the semi-arid climate. Nonetheless, physiological parameters remained within the comfort limits for pigs at this stage.

This study highlights the importance of simple, automated technologies with high scalability potential to improve the environmental conditions of finishing pigs in semi-arid regions, directly impacting daily weight gain and productivity. Future research should incorporate expanded experimental designs with additional animal pens to properly evaluate feed conversion efficiency under different thermal management strategies. Studies should also systematically assess varying ventilation intensities and microsprinkler application schedules to determine optimal combinations for thermal comfort optimization in production environments. Additionally, future investigations should include economic feasibility analyses, comparing implementation costs and potential benefits of the proposed prototype relative to commercial alternatives, to support broader adoption and informed decision-making by producers.

## Data Availability

The datasets generated during and/or analysed during the current study are available in the Figshare repository, 10.6084/m9.figshare.24857961.

## References

[CR54] Oliveira CP, Sousa FCD, Silva ALD, Schultz ÉB, Valderrama Londoño RI, Souza PARD (2025) Heat stress in dairy cows: impacts, identification, and mitigation strategies—a review. Animals 15(2):249. 10.3390/ani15020249} 10.3390/ani15020249 PMC11758294 39858249

[CR1] Alves MDFA, Pandorfi H, Montenegro AADA, Silva RABD, Gomes NF, Santana TC, Silva WAD (2023) Evaluation of body surface temperature in pigs using geostatistics. Agriengineering 5:1090–1103. 10.3390/agriengineering5020069

[CR2] Alves MDFA, Pandorfi H, Soares RGF, Almeida GLPD, Santana TC, Silva MVD (2024) Computational techniques for analysis of thermal images of pigs and characterization of heat stress in the rearing environment. Agriengineering 6:3203–3226. 10.3390/agriengineering6030183

[CR3] Amaral PIS, Ferreira RA, Pires AV, da Silva Fonseca L, Gonçalves SA, de Souza GHC (2014) Performance, behaviour and physiological responses of finishing pigs under different lighting programs. J Anim Behav Biometeorol 2:54–59. 10.14269/2318-1265.v02n02a05

[CR4] Amorim MN, Turco SHN, Costa DS, Ferreira IJS, Silva WP, Sabino ALC, Silva-Miranda KO (2024) Discrimination of ingestive behavior in sheep using an electronic device based on a triaxial accelerometer and machine learning. Comput Electron Agric 218:108657. 10.1016/j.compag.2024.108657

[CR5] Aragona F, Rizzo M, Arrigo F, Arfuso F, Fazio F, Giudice E, Pugliatti P, Piccione G, Giannetto C (2025) Pilot study: simultaneous daily recording of total locomotor activity and heart rate in horses for application in precision livestock farming. Animals 15:1189. 10.3390/ani15091189 40362004 10.3390/ani15091189 PMC12070940

[CR6] Araújo CDAP (2021) Performance of pigs in the growth phase in Cariri Paraibano. Universidade Federal de Campina Grande. http://dspace.sti.ufcg.edu.br:8080/jspui/handle/riufcg/19148. Accessed 12 November 2024

[CR7] Araújo Júnior GDNA, Queiroz MG, Jardin AMDRF, Silva MJ, Pereira PC, Silva TGF (2019) Characterization of the predominant direction, maximum and average speed of the wind of the municipality of Petrolina-PE. Pensar acadêmico 17:43–49. 10.21576/pa.2019v17i1.363

[CR8] Associação Brasileira dos Criadores de Suínos - ABCS (2024) Retrato da Suinocultura Brasileira. ABCS Notícias, Matrizes Suínas tecnificadas no Brasil, por região e por modelo de produção em 2023, p. 06. 2024. https://abcs.org.br/wp-content/uploads/2024/04/Retrato-da-Suinocultura-2024-Web.pdf. Accessed 12 November 2024

[CR9] Associação Brasileira de Proteína Animal. – ABPA (2024) Relatório anual 2024. p. 76. https://abpa-br.org/wp-content/uploads/2024/04/ABPA-Relatorio-Anual-2024_capa_frango.pdf. Accessed 12 November 2024

[CR10] Associação Brasileira de Proteína Animal. – ABPA (2023) Relatório anual 2023. p. 73. https://abpa-br.org/wp-content/uploads/2023/04/Relatorio-Anual-2023.pdf. Accessed 12 November 2024

[CR11] Barnabé H, Pandorfi NF, Gomes GL, Ameida C, Guiselin I (2020) Performance and welfare of finishing pigs subjected to climatecontrolled environments and supplementary lighting. Eng Agric 40:294–302. 10.1590/1809-4430-Eng.Agric.v40n3p294-302/2020

[CR12] Bernabucci G, Evangelista C, Girotti P, Viola P, Spina R, Ronchi B, Bernabucci U, Basiricò L, Turini L, Mantino A, Mele M, Primi R (2025) Precision livestock farming: an overview on the application in extensive systems. Ital J Anim Sci 24:859–884. 10.1080/1828051X.2025.2480821

[CR13] Buffington DE, Collazo-Arocho A, Canton GH, Pitt D, Thatcher WW, Collier RJ (1981) Black globe-humidity index (BGHI) as comfort equation for dairy cows. Trans ASAE 24:711–0714. 10.13031/2013.34325

[CR14] Castro Júnior SL, Silva IJO (2021) Specific air enthalpy as an indicator of thermal stress in livestock animals. Int J Biometeorol 65:149–161. 10.1007/s00484-020-02022-8 32968875 10.1007/s00484-020-02022-8

[CR15] Cunha APM, Alvalá RC, Nobre CA, Carvalho MA (2015) Monitoring vegetative drought dynamics in the Brazilian semiarid region. Agric Meteorol 214:494–505. 10.1016/j.agrformet.2015.09.010

[CR16] Dias CA, Carraro BZ, Dallanora D, Coser FJ, Machado GS, Machado IP, Pinheiro R, Rohr SA (2011) Manual Brasileiro de Boas práticas Agropecuárias Na Produção de suínos. Embrapa Suínos e Aves, Brasilia, DF: ABCS, MAPA, Concordia

[CR17] Diniz CDDSC, Ataíde EM (2023) Different substrates in the germination of pomegranate seeds. Braz J Anim Meio Ambiente Res 6:1876–1882. 10.34188/bjaerv6n2-073

[CR18] Ferreira RA, Fassani EJ, Ribeiro BPVB, Oliveira RD, Cantarelli VDS, Abreu MLT (2015) Lighting programs to growth pigs. Arch Vet Sci 20:65–70

[CR19] Giannetto C, Aragona F, Fazio F, Piccione G, Giudice E, Arfuso F, Zumbo A (2025) Investigation of the impact of seasonal climate conditions on feed intake and body weight in horses. Int J Biometeorol 69:1101–1110. 10.1007/s00484-025-02881-z 40009160 10.1007/s00484-025-02881-z

[CR20] Giannetto C, Cerutti RD, Scaglione MC, Fazio F, Aragona F, Arfuso F, Zumbo A, Piccione G (2022) Simultaneous recording of subcutaneous temperature and total locomotor activity in *Bos taurus* and *Bos indicus* raised in a subtropical region of Argentina. Trop Anim Health Prod 54:371. 10.1007/s11250-022-03365-7 36326987 10.1007/s11250-022-03365-7

[CR21] Godyń D, Herbut P, Angrecka S, Corrêa Vieira FM (2020) Use of different cooling methods in pig facilities to alleviate the effects of heat stress—a review. Animals 10(9):1459. 10.3390/ani10091459 32825297 10.3390/ani10091459 PMC7552673

[CR22] Gomes NF, Pandorfi H, Barnabé JMC, Guiselini C, Almeida GLPD, Holanda MCRD, Holanda AC, Silva MVD (2021) Behavior of pigs subjected to climate control system in the semi-arid region of Pernambuco. Braz Dyn 88:34–38. 10.15446/dyna.v88n218.93887

[CR23] Jeppsson KH, Olsson AC, Nasirahmadi A (2021) Cooling growing/finishing pigs with showers in the slatted area: effect on animal occupation area, pen fouling and ammonia emission. Livest Sci 251:104607. 10.1016/j.livsci.2021.104607

[CR24] Kiefer C, Moura MSD, Silva EAD, Santos APD, Silva CM, Luz MFD, Nantes CL (2010) Response of finishing swine maintained in different thermal environments. Rev Bras Saude Prod Anim 11:496–504

[CR25] Knox RV (2025) Worldwide perspective for swine production and reproduction for the next 20 years. Theriogenology 234:24–33. 10.1016/j.theriogenology.2024.11.020 39631253 10.1016/j.theriogenology.2024.11.020

[CR26] Lovarelli D, Bovo M, Giannone C, Santolini E, Tassinari P, Guarino M (2024) Reducing life cycle environmental impacts of milk production through precision livestock farming. Sustain Prod Consum 51:303–314. 10.1016/j.spc.2024.09.021

[CR27] Lykhacha A, Lykhachb V, Mylostyvyic R, Barkard Y, Shpetnye M, Izhboldinaf O (2022) Influence of housing air temperature on the behavioural acts, physiological parameters and performance responses of fattening pigs. J Anim Behav Biometeorol 10:2226. 10.31893/jabb.22026

[CR28] McManus C, Tanure CB, Peripolli V, Seixas L, Fischer V, Gabbi AM, Menegassi SRO, Stumpf MT, Kolling GJ, Dias E, Costa JBG Jr (2016) Infrared thermography in animal production: an overview. Comput Electron Agric 123:10–16. 10.1016/j.compag.2016.01.027

[CR29] Mellado M, Gaytán L, Macías-Cruz U, Avendaño L, Meza-Herrera C, Lozano EA, Rodríguez Á, Mellado J (2018) Effect of climate and insemination technique on reproductive performance of gilts and sows in a subtropical zone of Mexico. Aust J Vet Sci 50:27–34. 10.4067/S0719-81322018000100106

[CR30] Moi M, Nääs IDA, Caldara FR, Paz ICDL, Garcia RG, Cordeiro AF (2014) Vocalization data mining for estimating swine stress conditions. Eng Agric 34:445–450. 10.1590/S0100-69162014000300008

[CR31] Moreira MDR, Trabachini A, Amorim MDN, Harada ÉDS, da Silva MA, Silva-Miranda KOD (2024) The perception of Brazilian livestock regarding the use of precision livestock farming for animal welfare. Agriculture 14:1315. 10.3390/agriculture14081315

[CR32] Nazareno AC, Silva IJ, Nunes ML, Castro ACD, Miranda KO, Trabachini A (2012) Bioclimatic characterization of outdoor and confined systems for pregnant sows. Rev Bras Eng Agri Ambient 16:314–319. 10.1590/S1415-43662012000300013

[CR33] Norton T, Chen C, Larsen MLV, Berckmans D (2019) Precision livestock farming: building ‘digital representations’ to bring the animals closer to the farmer. Animal 13:3009–3017. 10.1017/S175173111900199X 31516101 10.1017/S175173111900199X

[CR34] Oliveira DCG, Di Campos MS, Passé-Coutrin N, Potiron CO, Bilba K, Arsène MA, Junior HS (2021) Modeling of the thermal performance of piglet house with non-conventional floor system. J Build Eng 35:102071. 10.1016/j.jobe.2020.102071

[CR35] Oliveira DCG, Moi M, Nakanishi EY, Sousa MS, Martello LS, Savastano Júnior H (2016) Pig performance in relation to different cooling systems. International Symposium on Ambience and Engineering in Sustainable Animal Production: Hot and Temperate Climate Conditions – SIAPAS. Viçosa: Federal University of Viçosa

[CR36] Onset, HOBO Datalogger Temp/RH/2 ext, Channels (U12-0011). https://www.onsetcomp.com. Accessed 02 October 2024

[CR37] Papakonstantinou GI, Voulgarakis N, Terzidou G, Fotos L, Giamouri E, Papatsiros VG (2024) Precision livestock farming technology: applications and challenges of animal welfare and climate change. Agriculture 14:620. 10.3390/agriculture14040620

[CR38] Pereira CB, Dohmeier H, Kunczik J, Hochhausen N, Tolba R, Czaplik M (2019) Contactless monitoring of heart and respiratory rate in anesthetized pigs using infrared thermography. PLoS One 14:e0224747. 10.1371/journal.pone.0224747 31693688 10.1371/journal.pone.0224747 PMC6834247

[CR39] Perissinotto M, Moura DJ, Cruz VF, Souza SRLD, Lima KAOD, Mendes AS (2009) Thermal comfort on subtropical and mediterranean climate analyzing some physiological data through fuzzy theory. Cienc Rural 39:1492–1498. 10.1590/S0103-84782009005000094

[CR40] Possagnolo BO, Condotta ICFS, Amorim MDN, Harada ÉDS, Piedade SMS, Cipriano DAM, Silva-Miranda KO (2024) Recyclable rubber flooring in farrowing crates and its influence on the development and welfare of piglets in the maternity phase. Agriculture 14(6):807. 10.3390/agriculture14060807

[CR41] Renaudeau D, Dourmad JY (2022) Future consequences of climate change for European Union pig production. Animal 16:100372. 10.1016/j.animal.2021.100372 34690100 10.1016/j.animal.2021.100372

[CR42] Ricci GD, da Silva-Miran KO, Titto CG (2019) Infrared thermography as a non-invasive method for the evaluation of heat stress in pigs kept in pens free of cages in the maternity. Comput Electron Agric 157:403–409. 10.1016/j.compag.2019.01.017

[CR43] Rigo EJ, Nascimento MRB, Silva NAM (2019) Performance and thermoregulation of lactating sows housed in different locations inside a shed with an evaporative cooling system in a tropical environment. Arq Bras Med Vet Zootec 71:1750–1758. 10.1590/1678-4162-11370

[CR44] Rodrigues VC, Silva IJO, Vieira FMC, Nascimento ST (2011) A correct enthalpy relationship as thermal comfort index for livestock. Int J Biometeorol 55:455–459. 10.1007/s00484-010-0344-y 20607305 10.1007/s00484-010-0344-y

[CR45] Santos JHT, Tinôco IFF, Costa CA (2012) Evaluation of ventilation systems in swine finishing shed under conditions of Central–Western Brazil. Reveng 20:201–209. 10.13083/reveng.v20i3.270

[CR46] Santos SA, Correia MF, Aragão MRS, Oliveira MB, Santos EP (2011) Análise das trocas de água, energia e CO_2_ em área de caatinga. Cienc Nat 33:147. 10.5902/2179460X9406

[CR47] Saputro AD, Yantidewi M (2021) Analysis of air temperature and humidity in kedunggalar against BMKG data based on DHT11 sensor. Journal of Physics: Conference Series 1805:012045. 10.1088/1742-6596/1805/1/012045

[CR48] Silva WA, Pandorfi H, Vigoderis RB, Almeida GLP, Moraes AS, Guiselini C, Silva MV, Marinho GTB (2023) Exploratory inference of the ingestive behavior of pigs in the growth phase in an air-conditioned environment. Livest Sci 271:105232. 10.1016/j.livsci.2023.105232

[CR49] Soerensen DD, Pedersen LJ (2015) Infrared skin temperature measurements for monitoring health in pigs: a review. Acta Vet Scand 57:1–11. 10.1186/s13028-015-0094-2 25644397 10.1186/s13028-015-0094-2 PMC4337315

[CR50] Souza RG, Gomide APC, Feitosa TJO, Crispim EG, Leite DPSBM, França VS, Sousa GR (2020) Influence of temperature on swine maternity: bibliographic review. Res Soc Dev 9:e193932757. 10.33448/rsd-v9i3.2757

[CR51] Thom EC (1959) The discomfort index. Weatherwise 12:57–61. 10.1080/00431672.1959.9926960

[CR52] Trabachini A, Dias CDS, Mathias MRM, Wen TC, Caneppele FL, Harada ÉS, Amorim MN, Miranda KOS (2024) Automation to improve pig welfare using fuzzy logic. Rev Bras Cienc Agrar 19:1–10. 10.5039/agraria.v19i3a3532

[CR53] Zhang M, Wang X, Feng H, Huang Q, Xiao X, Zhang X (2021) Wearable internet of things enabled precision livestock farming in smart farms: a review of technical solutions for precise perception, biocompatibility, and sustainability monitoring. J Clean Prod 312:127712. 10.1016/j.jclepro.2021.127712

[CR55] Oliveira CP, Sousa FCD, Silva ALD, Schultz ÉB, Valderrama Londoño RI, Souza PARD (2025) Heat stress in dairy cows: impacts, identification, and mitigation strategies—a review. Animals 15(2):249. 10.3390/ani15020249} 10.3390/ani15020249 PMC11758294 39858249

